# Electrocardiogram (EKG) knowledge and interpretation proficiency among healthcare professionals in Pakistan: a prospective cross-sectional study

**DOI:** 10.1097/MS9.0000000000002220

**Published:** 2024-06-17

**Authors:** Zair Hassan, Usha Kumari, Maria Waseem, Uzair Yaqoob, Moiz Sahito, Syeda Arshia Zehra, Nikhil Reddy, Muneeb Ullah Jan, Salim Surani, Aarash Khan

**Affiliations:** a Afridi Medical Complex and Teaching Hospital, Peshawar; b Lady Reading Hospital, Peshawar; c Dow University of Health Sciences; d Shaheed Mohtarma Benazir Bhutto Institue of Trauma, Karachi, Pakistan; e Kakatiya Medical College, Telangana, India; f Adjunct Clinical Professor, Texas A&M University, Research Collaborator, Mayo Clinic, Rochester, MN, USA; g Herat University, Herat, Afghanistan

**Keywords:** 12-lead EKG, cardiac monitoring, cardiac rhythm, cardiovascular disease, efficient screening, electrocardiography

## Abstract

**Background:**

Electrocardiogram (EKG) is a commonly used diagnostic tool for the evaluation of the electrical activity of the heart. The purpose of this study was to assess the knowledge and interpretation proficiency of EKG among healthcare professionals (HCPs) in Pakistan.

**Methods:**

This prospective cross-sectional study was conducted among HCPs working in different healthcare settings. A structured questionnaire was used to assess the participants’ theoretical knowledge and ability to interpret EKG findings. The data were analyzed using descriptive statistics and χ^2^ tests. The study indicates that EKG knowledge and interpretation proficiency among HCPs in Pakistan is unsatisfactory. The inadequacy of training periods of EKG training sessions and insufficient participation of HCPs in offered training opportunities put forward the need for the formation and introduction of better structured and efficient EKG training programmes.

**Results:**

A total of 511 HCPs participated in the study, 28% of whom reportedly had received formal training for EKG interpretation. About 80% of the participants correctly identified theoretical questions pertaining to EKG, while 58% of the participants were able to accurately interpret EKG findings, and most HCPs (69.9%) read fewer than ten EKGs per week.

**Conclusion:**

This study demonstrates a lack of expertise and a poor understanding of EKG in HCPs of Pakistan. The low level of EKG knowledge and interpretation proficiency among HCPs may lead to diagnostic errors and poor patient outcomes. Therefore, efforts should be made to improve EKG education and training among HCPs in Pakistan.

## Introduction

Highlights

- The study puts forward the proficiency of medical students, postgraduate trainees, and healthcare physicians of Pakistan in the interpretation of electrocardiogram (EKG); the initial thus most commonly employed choice of investigation for several cardiac conditions.
- Most opted mode of learning EKG interpretation was found to be a discussion with peers and seniors amongst healthcare professionals, while participation in formal EKG training programs was reported to be insufficient.
- The maximum training period for formal EKG training sessions was reported to be less than a week by those who participated.
- Knowledge of EKG is found to be limited in most Healthcare Professionals of Pakistan indicating a need for better and structured formal EKG training.

Electrocardiogram (EKG) is a gold-standard diagnostic tool commonly used to measure the heart’s electrical activity in patients with chest pain. It allows the specialist to assess the likelihood and nature of heart disease. It is non-invasive, simple to use, and cost-effective. The EKG is essential in modern medicine since it provides information on identifying acute coronary syndromes and cardiac arrhythmias while incorporating automated machine scanning simultaneously^1–4^. Healthcare professionals (HCPs) are required to make prompt decisions and follow adequately formed treatment plans based on the efficacy of diagnostic tools employed in a healthcare setting. EKG is a primary diagnostic tool used by physicians in the cardiac unit. Early detection and management of cardiac disease can be life-saving, which emphasizes the necessity of doctors’ competence in EKG interpretation. Non-cardiac specialists or doctors working in an emergency department (ED) or ICU, on the other hand, are found to have a high rate of EKG misinterpretation^4,5^. Delayed diagnosis, patient mismanagement and wastage of resources are all potential repercussions of poor EKG comprehension^6^, which can add to associated mortality and morbidity with increased poor patient outcomes.

ED physicians are typically the first to respond to cardiac arrest incidents in hospitals and should possess a fundamental understanding of resuscitation. EKG could be the only basis for the medical decision in the scenario. Therefore, the need to recognize the most common EKG abnormalities (arrhythmia, ischemia or infarct) cannot be overstated. In Pakistan, where resources are scarce, this is particularly crucial. The correct finding that was most usually selected was ST-segment alterations or T-wave abnormalities^7^. The correct interpretation of an EKG among doctors varies greatly between 36 and 96%^8^.

If the ED physician can diagnose a patient timely with the use of EKG, it can be life-saving. It should be mentioned that interpreting EKG can be challenging and requires proper training and practice. The knowledge and EKG interpretation abilities of ED and ICU doctors has not been thoroughly evaluated in research. The literature supports the lack of EKG knowledge and interpretation difficulties among ED and ICU physicians^9^.

Most primary care and specialist physicians use EKG interpretation, a typical cognitive skill that graduates and residents develop during their training^10^, for the evaluation of patients presenting with chest discomfort. We conducted a preliminary study to evaluate EKG knowledge and interpretation among Pakistani ED doctors, ICU doctors, and ED residents. The results of this study will spur additional discussion among medical educators in Pakistan, which will ultimately result in an improvement in how EKG is used to evaluate patients in general practice.

It is noticed that the knowledge and interpretation of EKG are suboptimal among physicians in Pakistan. It has been proposed that more precise EKG interpretations can be achieved by studying more in-depth information and enhancing the interpretation of EKG sessions in general practice.

## Materials and methods

This cross-section descriptive study was conducted between January and September 2020 in five different tertiary care hospitals in Pakistan. The selection of these hospitals was based on the investigator’s location at the time of the study. Informed and written consent was obtained from each participant after explaining the purpose of the study. They were also instructed on how to complete the questionnaire. In the context of our research paper titled “Electrocardiogram (EKG) Knowledge and Interpretation Proficiency Among Healthcare Professionals in Pakistan,” we used the Delphi method to develop a questionnaire to assess the knowledge and interpretation proficiency of EKG among healthcare professionals in Pakistan. We started by identifying a panel of experts who have knowledge and experience in EKG interpretation and healthcare in Pakistan. The experts included cardiologists, electrophysiologists, nurses, and other healthcare professionals who have experience working with EKGs in Pakistan. The initial questionnaire was developed based on the literature review, and it included questions related to the demographic characteristics of the participants, their EKG knowledge, and their interpretation proficiency. The questions were open-ended and allowed for multiple answers. We distributed the initial questionnaire to the panel of experts and asked them to provide their responses. We encouraged them to add or modify questions based on their experience and knowledge. We collected and analyzed the responses to the initial questionnaire. We also identified the areas where there was consensus among the experts and the areas where there was disagreement. We used this information to revise the questionnaire and develop a new version. Then, we repeated the process by distributing the revised questionnaire to the panel of experts and collecting their responses. Continuing this process until there was a consensus among the experts on the final version of the questionnaire. Once the final version of the questionnaire was developed, it was validated using a pilot study. The pilot study included a small sample of healthcare professionals in Pakistan, and the results were used to evaluate the reliability and validity of the questionnaire. Finally, we used the validated questionnaire in our research to assess the EKG knowledge and interpretation proficiency of healthcare professionals in Pakistan. The first section consisted of demographic data. The second section consisted of true/false questions regarding EKG knowledge, and the final section included multiple-choice questions (MCQs) about the EKG interpretation practice. The MCQs comprised a short cardiology-related scenario, the EKG strips, and four options for each question. The EKG strips that were used in the questionnaire were discussed with two cardiologists separately. Participation in the study was voluntary. Ethical approval was obtained from the Institution Review Board before conducting the research. The data were analyzed using the Statistical Package for the Social Sciences (SPSS) software, version 22.0. Knowledge questions were assessed using descriptive statistics like percentage and frequency. *P* value less than 0.05 was statistically significant.

## Results

**Figure 1:**
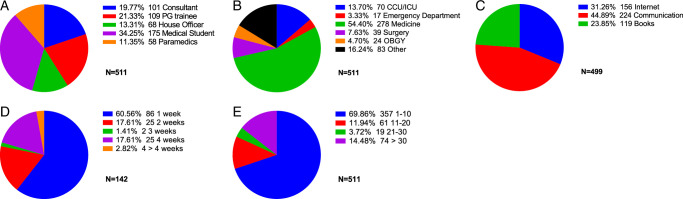
Frequency of HCPs according to (A) designation, (B) department, (C) method of learning, (D) duration of the training, (E) the number of electrocardiograms read per week. (N=Frequency). HCP, healthcare professional.

Out of a total of 511 participants, 83% were female, and 17% were male, with the majority (77.9%) being under 30 years old. Most of the population were medical students, followed by postgraduate trainees (21.3%) and physicians (19.8%). Discussion with their peers and seniors served as their preferred mode of learning for most of them (34.2%), who belonged to the general medicine department (43.8%). 72.2% of participants had never participated in a formal EKG training session. The maximum training period for those who participated (27.8%) was less than one week. Most of the HCPs (69.9%) said they read fewer than 10 EKGs every week. The details of these characteristics are given in Figure 1.

**Figure 2:**
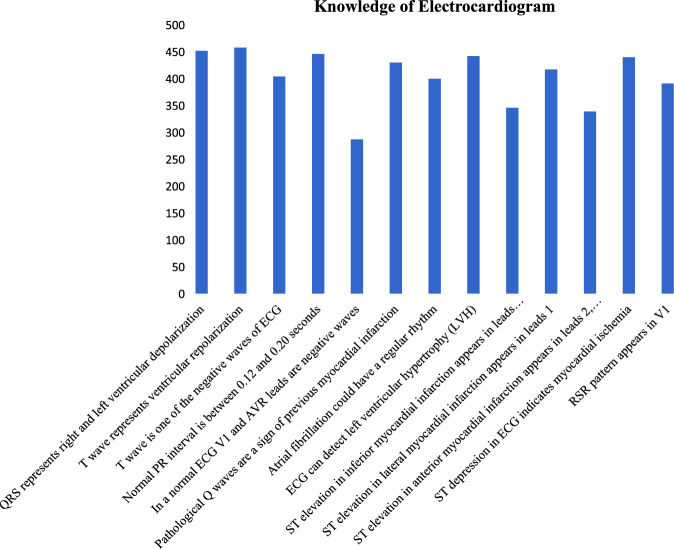
Frequency of each of the correct electrocardiogram knowledge items (*N*=511).

**Figure 3:**
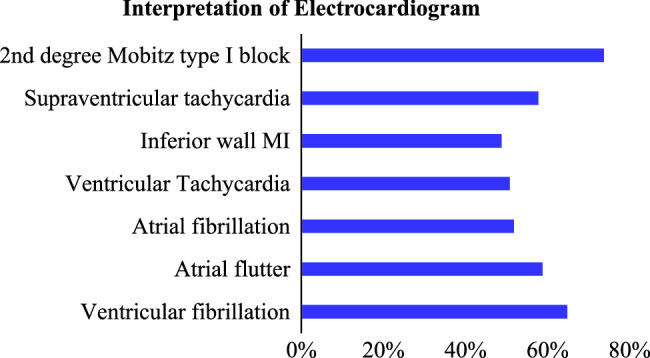
Percentage of each of the correct electrocardiogram interpretation items (*N*=511).

**Figure 4:**
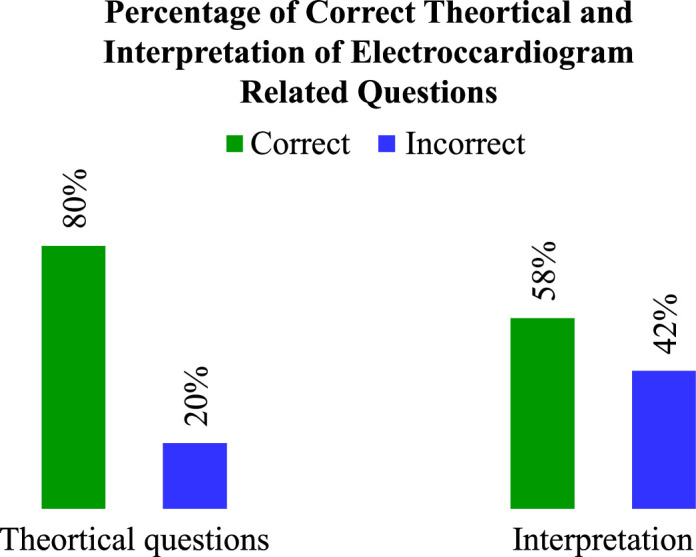
Uses a strips electrocardiogram to show the percentage of total correct and incorrect theoretical questions (Knowledge questions, *n*=6649) and the scenarios from real life (Interpretation questions, *n*=3577).

For various theoretical knowledge, the most correctly answered EKG knowledge item was “T wave represents ventricular repolarization” and the least correctly answered was found to be “In a normal ECG, V1 and augmented vector right leads are negative waves”. The frequency of correct individual EKG knowledge items is presented in Figure 2 while Figure 3 shows the percentage of each correct practical EKG interpretation item. The stratification of correct EKG knowledge and interpretation based on designations, departments, and chosen learning methods revealed statistically significant associations (*P* 0.05), as shown in Table 1, whereas percentage of total correct and incorrect theoretical questions of EKG has been shown in Figure 4. Age groups exhibited statistical insignificance (*P*>0.05) with knowledge questions but significance with EKG interpretation (*P*<0.05), whereas sex and EKG training showed statistically insignificant (*P*>0.05) results.

**Table 1:** Knowledge and interpretation of various EKG patterns in HCPs of different designations, departments, and specialties.

	Designation					Department						Method of learning		
Knowledge and interpretation items	Physician	Resident	HO/intern	MS	Nurse	CCU/ICU	ER.	Medicine	Surgery	OBGY	Other	Internet	Institute	Books
QRS represents right and left ventricular depolarization*,**,***	95	103	61	152	40	61	12	255	33	17	75	129	198	117
T wave represents ventricular repolarization*,**,***	96	108	57	157	39	56	08	266	38	24	67	130	200	118
T wave is one of the negative waves of EKG*,**,***	95	97	59	115	37	59	15	232	27	16	56	107	185	105
Normal PR interval is between 0.12 and 0.20 sec***	91	98	60	151	46	60	15	245	32	23	72	132	191	114
In a normal EKG V1 and AVR leads are negative waves*,***	69	80	26	81	30	43	12	166	16	12	39	81	119	86
Pathological Q waves are a sign of previous MI *,**,***	95	104	41	149	40	62	10	241	28	21	69	123	189	110
Atrial fibrillation could have a regular rhythm*,***	90	95	45	128	42	57	10	215	33	16	70	110	176	109
EKG can detect left ventricular hypertrophy*,***	100	105	51	153	33	60	14	246	36	16	71	126	193	114
ST elevation in inferior MI appears in leads V1–V6*,**,***	91	97	37	95	26	55	11	205	19	11	46	95	146	100
ST elevation in lateral MI appears in leads 1^,^ AVL^,^ V5^,^ and V6*,**,***	95	100	49	130	43	61	12	237	30	17	61	127	172	108
ST depression in EKG indicates myocardial ischemia*	93	103	57	146	41	65	13	236	33	19	75	130	192	108
RSR pattern appears in V1^,^ V2^,^ and V3 in the right bundle branch block*,**,***	86	96	50	119	40	60	15	202	28	16	71	114	163	106
Ventricular fibrillation*,**,***	90	84	31	99	27	50	09	199	17	12	46	23	28	19
Atrial flutter*,**,***	87	90	20	85	15	48	09	169	17	12	44	64	115	85
Atrial fibrillation*,**,***	74	80	18	68	24	48	09	138	11	13	47	48	09	138
Ventricular tachycardia*,**,***	75	74	23	67	22	50	12	137	11	10	43	74	101	84
Inferior wall myocardial infarction*,**	70	65	22	69	24	24	10	148	16	06	47	76	109	64
Supra-ventricular tachycardia*,**,***	77	90	31	80	15	43	07	162	21	14	47	69	127	93
Second-degree Mobitz type I block*,**,***	92	87	40	127	28	48	05	220	26	17	60	103	174	92

AVR: augmented vector right, AVL: augmented vector left CCU, critical care unit; EKG, electrocardiogram; ER, emergency department; HCP, healthcare professional; HO, house officer; MI, myocardial infarction; MS, medical student; OBGY, obstetrics, and gynecology.

*P < 0.05 for designation.

**P < 0.05 for department.

***P < 0.05 for method of learning.

## Discussion

EKG is one of the most frequently utilized diagnostic modalities in healthcare settings, and its importance in diagnosing cardiac conditions cannot be overstated. While other tests such as troponin levels may be more accurate in emergencies, EKG remains the most common diagnostic tool for individuals with chest discomfort^11^. The ability to record and interpret an EKG is therefore essential for HCPs in high-risk areas to be able to intervene quickly. Several studies conducted in the last five years have highlighted the importance of EKG knowledge and interpretation proficiency among HCPs^12^. To the best of our knowledge, this is the first study to evaluate the comprehension and interpretation abilities of medical students and HCPs. The study’s questions were carefully chosen to convert participants’ understanding of EKG into quantifiable results. This study puts forward that most of the participants never employed a formal EKG training program and general healthcare professionals preferred learning from their peers as a means of keeping up with their EKG interpretation skills. Most of EKG training programs were found to be of inadequate duration that is of less than a week with no standardized examination criterion to estimate EKG interpretation proficiency.

A descriptive cross-sectional study conducted among medical students and HCPs of Ardabil University, Iran in 2022 compares with our study and shows that HCPs, including physicians and nurses, struggled with interpreting complex EKGs especially those involving arrhythmias and conduction abnormalities. The study also noted that additional training and education could improve EKG interpretation accuracy^13^. A similar study found that doctors with greater clinical experience and more experience in reading EKGs answered more questions correctly than their younger colleagues who had lesser experience and opportunities of reading EKGs^14^. In addition, a quasi-experimental study carried out in 2021 revealed that nurses who received EKG education and training had higher EKG interpretation scores than those who did not. The study highlighted the importance of EKG interpretation proficiency among emergency medicine residents, as misinterpretation of EKGs could lead to significant medical errors^15^. Our study found remarkable findings in the interpretation of atrial fibrillation, ventricular tachycardia (VT), supra-ventricular tachycardia (SVT), inferior wall myocardial infarction (IWMI), myocardial ischemia, ventricular repolarization and right bundle branch block (RBBB) amongst healthcare workers. It was shown that half of the participants were unable to identify atrial fibrillation^16^. Improper computerized interpretation of atrial fibrillation and physician’s failure to correct the faulty reading can lead to unnecessary and potentially harmful medical procedures as well as inappropriate use of medical resources. Together with this, the use of an automated algorithm to screen for atrial fibrillation resulted in a high rate of false positives and unnecessary referrals to cardiology according to another study^17^. Moreover, computer-aided diagnosis system for atrial fibrillation was found to have high sensitivity but low specificity, leading to overdiagnosis and over-treatment^18–20^.

The frequency and percentage of each EKG interpretation item have been shown in Figures 3 and 4, respectively. Up to 48.5% of participants in our study were unable to recognize VT. Research shows that STEMI and VT are the most common conditions mimicked by artifacts^21^. Similarly, despite using a criteria-based approach, physicians in another study were unable to distinguish between supra-ventricular-wide complex tachycardia and VT^22^. Additionally, 42.5% of the participants were unable to identify SVT. SVT is a type of tachycardia that causes heart rates to exceed 100 beats per min and has an electro-pathologic substrate that occurs above the bundle of His^23^. A high index of suspicion is needed to diagnose cardiomyopathy because prolonged and consistently increased heart rates brought on by various types of SVT have been associated with the condition^24^. When adjusting for age and gender, SVT is expected to have a 35 per 100 000 person-year incidences^25^. The severity of SVT symptoms depends on several factors, including the patient’s age, the existence of heart and lung illness, the duration of SVT episodes, and others. Patients might only have minor symptoms or be asymptomatic, which could cause the diagnosis to be delayed^23^. The patient in our example had symptoms. Thus, the diagnosis should have been obvious. Differential diagnosis must take sinus tachycardia into account. Episodic SVT is frequently misdiagnosed as anxiety or panic disorder, especially in patients with a psychiatric illness^26^ resulting in delayed treatment and diagnosis. The question about IWMI was inaccurately answered by nearly half of our participants. IWMI is often characterized by ST-segment elevation (STEMI) in II, III, and aVF leads in the EKG. Isolated anterior wall MI may not result in reciprocal changes and may lead to misunderstandings in contrast to inferior MI, where reciprocal changes are frequently observed^24^. The existence of these patterns in patients who present with chest pain may offer a challenge to primary care physicians and result in a potential delay in the treatment of MI^27^.

The most accurate answers, however, were for the statement, “T wave represents ventricular repolarization.” Earlier research on nurses produced less accurate answers to the T-wave question, which contrasts with the current study^28^. 86.3% of participants correctly identified the association between ST-segment depression and myocardial ischemia. This is a good indicator since it shows that medical students and healthcare workers in Pakistan have a solid understanding of MI and can correctly identify it using EKG. In a study conducted in England and Wales, the researchers found that more than a third of the 564 412 patients with STEMI or non-ST elevation MI (NSTEMI) were misdiagnosed in the hospital. Initial misdiagnosis of STEMI and NSTEMI was more likely in women, older patients, and those with comorbidities^29^. The study discovered that individuals who had an initial misdiagnosis were less likely to receive the recommended care and compared to those who received a right initial diagnosis, had considerably greater death rates^29^.

Only a quarter of participants were able to correctly interpret the rSr’ pattern and associate it with RBBB. An rSr’ pattern in the right precordial lead with a width less than 100ms is usually classified as an incomplete RBBB, which can be caused by high-placed leads (V1–V2) but also can be present in Brugada Syndrome^30^. A complete right bundle branch block (RBBB), according to the Minnesota Code, is defined as a QRS duration of more than 0.12sec, a secondary R wave in V1–V2 with T-wave inversions and a wide slurred S wave in lead 1, V5, and V6^31^.

Better outcomes in terms of patient care and management shall be encountered with the inoculation of formal EKG interpretation training programs with well-defined curriculum and clear learning objectives to be fulfilled corresponding to respective learning requirements of undergraduate and postgraduate healthcare professionals together with standardized examination criterion. Introduction of such training programs can significantly improve EKG interpretation proficiency amongst HCPs of Pakistan while mitigating the gap between theoretical understanding of EKG and real time EKG interpretation.

### Study limitations

The use of a structured questionnaire may not fully capture participants’ actual knowledge and skills in EKG interpretation. Mode of training, qualification, experiences, designations and differences of EKG interpretation proficiency between different departments of the HCPs have not been extrapolated and explored in detail in this study. In addition, the influence of individual demographic characteristics of participants such as age, sex and professional status on study outcomes has not been considered. Most of the participants in our study were medical students, and their understanding of the EKG was limited with no subsequent evaluation of how well the participants retained the information. Together with this, our study did not assess the long-term impact of knowledge of EKG on actual patient outcomes. Our study is pioneer in its nature, and further studies with rigorous research on the mentioned limitations based on our study are recommended.

## Conclusion

Most HCPs of Pakistan have limited knowledge of EKG indicating a need for more formal EKG training. The low interpretation proficiency of EKG among the participants suggests a need for additional training and support in this area. The use of a structured questionnaire and statistical analysis provides a reliable and objective method for assessing HCPs’ knowledge and skills in EKG interpretation. These findings highlight the importance of ongoing education and professional development for HCPs to improve the quality of patient care in Pakistan.

## Ethical approval

Protocol number: 206/LRH/MTI Ethical approval for this study was provided by the Ethical Review Board of Lady Reading Hospital Medical Teaching Institution, Peshawar, Pakistan on 02 March 2023.

## Consent

Written informed consent was obtained from the participants for publication and any accompanying images. A copy of the written consent is available for review by the Editor-in-Chief of this journal on request.

## Source of funding

Not applicable.

## Author contribution

Z.H.: idea conceptualization, study design. U.K.: study design, data collection, literature review, project administration, original draft writing. M.W.: writing original draft, literature review. U.Y.: data analysis. M.S.: writing original draft, literature review. S.A.Z.: data collection. N.R.: editing, project administration. M.U.J.: editing, data collection, reviewing. S.S.: proof reading, reviewing. A.K.: correspondence, reviewing.

## Conflicts of interest disclosure

The authors declare no conflicts of interest.

## Research registration unique identifying number (UIN)

UIN number: Researchregistry9452, Registry used: research registry, Hyperlink: https://www.researchregistry.com/browse-the-registry#home/.

## Guarantor

Zair Hassan.

## Data availability statement

Not applicable.

## Provenance and peer review

Not commissioned, externally peer-reviewed.
